# Crystal Structure, Photophysical Study, Hirshfeld Surface Analysis, and Nonlinear Optical Properties of a New Hydroxyphenylamino Meldrum’s Acid Derivative

**DOI:** 10.3390/molecules28052181

**Published:** 2023-02-26

**Authors:** Wulan Zeng, Xia Wang, Tao Zhou, Yunju Zhang

**Affiliations:** 1 Department of Chemistry, Chemical Engineering and Environmental Engineering, Weifang University, Weifang 261061, China; 2 Key Laboratory of Photoinduced Functional Materials, School of Chemistry and Chemical Engineering, Mianyang Normal University, Mianyang 621000, China

**Keywords:** photophysical properties, NBO, NLO properties, Hirshfeld surface analysis

## Abstract

The structural, photophysical, and vibrational properties of a new hydroxyphenylamino Meldrum’s acid derivative, 3-((2-hydroxyphenylamino)methylene)-1,5-dioxaspiro[5.5]undecane-2,4-dione (HMD), were studied. The comparison of experimental and theoretical vibrational spectra can help understand basic vibration patterns and provides a better interpretation of IR spectra. The UV–Vis spectrum of HMD was computed using density functional theory (DFT)/B3LYP/6-311 G(d,p) basis set in the gas state, and the maximum wavelength was in accord with the experimental data. The molecular electrostatic potential (MEP) and Hirshfeld surface analysis confirmed O(1)–H(1A)···O(2) intermolecular hydrogen bonds in the HMD molecule. The natural bond orbital (NBO) analysis provided delocalizing interactions between π→π* orbitals and n→σ*/π* charge transfer transitions. Finally, the thermal gravimetric (TG)/differential scanning calorimeter (DSC) and the non-linear optical (NLO) properties of HMD were also reported.

## 1. Introduction

It is known that Meldrum’s acid is an important organic reagent for the Knoevenagel condensation owing to its activated methylene structure. It has become a versatile building block and valuable intermediate for the synthesis of organic six-membered heterocycles [1,2,3,4,5]. In addition, Meldrum’s acid and its derivatives have also attracted much attention due to their pharmacological and biological properties, such as antioxidant [6,7], anticancer [8], antimicrobial [9], antibiotic [10], and inhibitory [11] properties and herbicidal activity [12]. With this background in mind, we have previously reported several approaches to design and synthesize the diverse heterocyclic compounds using the Meldrum’s acid as the intermediate. For example, Meldrum’s acid or its derivatives reacted with aldehydes in conditions of ethanol or TEAF (V_triethylamine_:V_methanoic acid_ = 4:1)/DMF (N, N-dimethylformamide) [13,14,15], benzenamine (under conditions of N,N-dimethylformamide dimethyl acetal) [16,17], and 5,6-dimethyl-1H-benzoimidazole or imidazole (under conditions of trimethyl orthoformate) [18,19,20]. Our previous studies have mainly focused on the design of the synthetic methods and the analysis of crystal structures. Furthermore, to the best of our knowledge, theoretical calculations or Hirschfeld surface analyses of Meldrum’s derivatives containing 1,5-dioxaspiro[5.5] undecane-2,4-dione and hydroxyphenyl groups are rare.

As a continuation of our previous work, in this study, a new hydroxyphenylamino Meldrum’s acid derivative combining two different groups was synthesized. In addition, the structural, photophysical, vibrational, thermal, and nonlinear optical properties of the title compound were investigated. The calculated electronic absorption parameters of **HMD** by time-dependent density functional theory (TD-DFT) were compared with the experimental data, which were supported by each other. Finally, the MEP, NBO, Hirshfeld surface analysis, the highest occupied molecular (HOMO), and the lowest unoccupied molecular orbital (LUMO) analyses were studied.

## 2. Results and Discussion

### 2.1. Structural Characteristic for HMD

**Figure 1:**
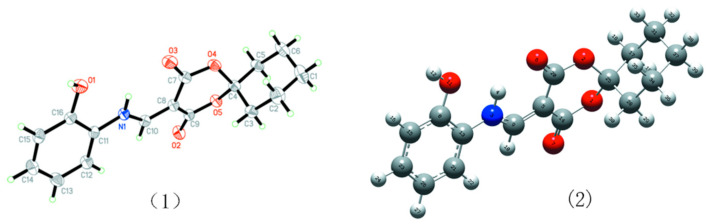
(**1**) *ORTEP* diagram of HMD. (**2**) Optimized structure of HMD.

The crystal structure of HMD disclosed by the X-ray analysis is shown in Table 1. The title compound adopted a linear conformation with 1,5-dioxaspiro[5.5]undecane-2,4-dione moiety and 2-hydroxyphenylaminomoiety via the C10 atom (Figure 1). The bond lengths of N(1)–C(10) and C(10)–C(8) were (1.321(3)Å) and (1.385(3) Å), respectively, which resembled the XRD data of the similar structure (1.330 Å and 1.396 Å) [21]. The bond angle of N(1)–C(10)–C(8) (125.70(2)°) was slightly smaller than that of the reported value (128.46°) [21]. The corresponding bond lengths (N(1)–C(10) and C(10)–C(8)) and the bond angle (N(1)–C(10)–C(8)) were calculated to be 1.333 Å, 1.380 Å, and 125.38°, respectively, which were in accord with the crystal data. Two planes (C8–C10/N1/O2/O5 and C7–C8/N1/O4) deviated from the plane of the 2-hydroxyphenylamino ring (C11–C16/O1/N1) with the dihedral angles (19.48(9)°) and (11.46(13)°), respectively. The torsion corresponding angles of C(7)–C(8)–C(10)–N(1) and C(9)–C(8)–C(10)–N(1) were −2.30(3)° and −175.60(2)°, respectively (Table 2).

**Figure 2:**
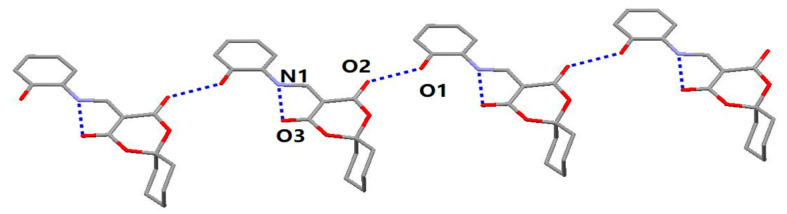
The 1D-chained structure for HMD.

**Figure 3:**
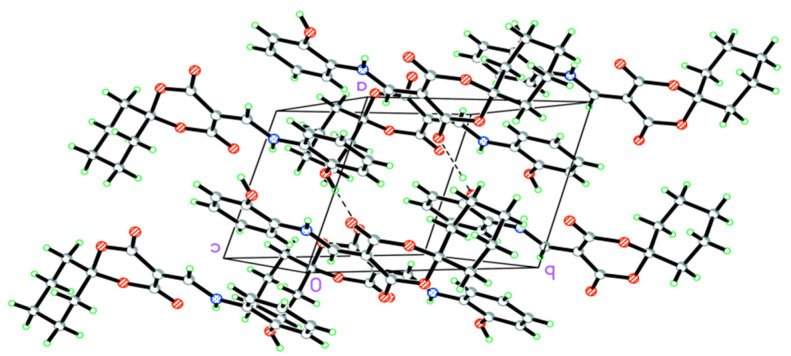
The packing arrangement of HMD.

As illustrated in Table 3, there were N(1)–H(1)···O(3) intramolecular interactions and O(1)–H(1A)···O(2) intermolecular interactions. As seen in Figure 2, the one-dimensional (1D) chained structure of HMD was connected by the O(1)–H(1A)···O(2) interhydrogen bonding, which played a vital role in helping to stabilize the packing in the crystal. The 3D supramolecular structure was linked by the above 1D chain (Figure 3).

### 2.2. IR Spectroscopy

**Figure 4:**
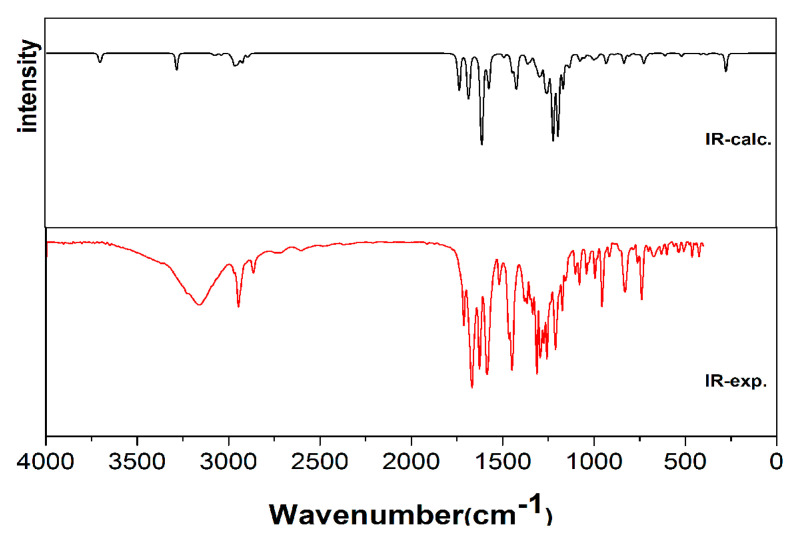
FT−IR and calculated IR for HMD.

Figure 4 depicts the calculated IR spectra using the DFT method and the basis set of the B3LYP/6-311G (d, p). To reduce the difference between the computational frequencies and the experimental values, 0.967 was used as the scaling factor. The DFT computation showed the O–H stretching modes at 3718 cm^−1^, which was consistent with the experimental and published values [15]. The N–H stretching peak seen at 3230 cm^−1^ was computed at 3297 cm^−1^. Peaks in the region 3100–3000 cm^−1^ were owing to the ν_C–H_ of the aromatic ring. In our study, the C–H stretching vibrations were calculated at 3056 cm^−1^ and 3098 cm^−1^. The CH_2_ stretching peaks of the 1,3-dioxane group were shown at 2943 cm^−1^ and 2862 cm^−1^. These peaks were computed from 2993 cm^−1^ to 2904 cm^−1^. For the carbonyl group, two strong bands appeared at 1718 cm^−1^ and 1670 cm^−1^ in the IR spectra and were calculated at 1744 cm^−1^ and 1691 cm^−1^. The peaks at 1625 cm^−1^, 1585 cm^−1^, and 1443 cm^−1^ were attributed to the C=C stretching modes. The corresponding peaks were calculated at 1618 cm^−1^, 1580 cm^−1^, and 1427 cm^−1^. The experimental spectrum of HMD showed C–O stretching vibrations at 1257 cm^−1^ and 1175 cm^−1^, while the calculated values were 1228 cm^−1^ and 1171 cm^−1^, which agreed with similar structural reports [16,17,21].

### 2.3. Electronic Analysis

**Figure 5:**
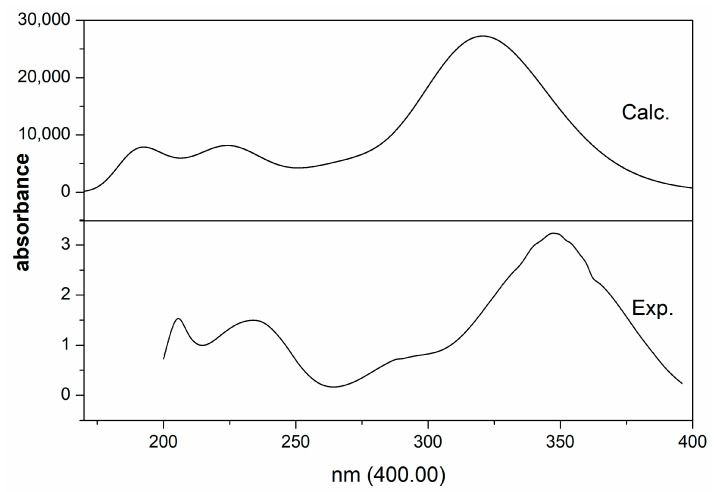
Experimental and calculated UV−Vis spectra for HMD.

Figure 5 depicts the UV–Vis absorption spectrum from 180 nm to 400 nm, along with the calculated value. The theoretical UV–Vis spectrum was obtained using the TD-DFT methods with the same basis set, and their excitation energies included the ten excited states. The oscillator strengths, wavelengths, and electronic transition orbits are listed in Table 4. As seen in Figure 5, three prominent bands appeared at the experimental/theoretical absorption spectra. The first band at 206/191 nm was dominated by 79HOMO−1→83LUMO+2 (40.64%) with oscillator strengths of 0.1113. The second band at 234/226 nm was contributed by the electronic transition 80HOMO→83LUMO+2 (48.30%). The third band at 346/321 nm was contributed by the electronic transition 80HOMO→81LUMO (97.0%) with the oscillator strengths of 0.6683, indicating π→π* and n→π* transitions of the molecule. These results were in accordance with previous research [16,17].

**Figure 6:**
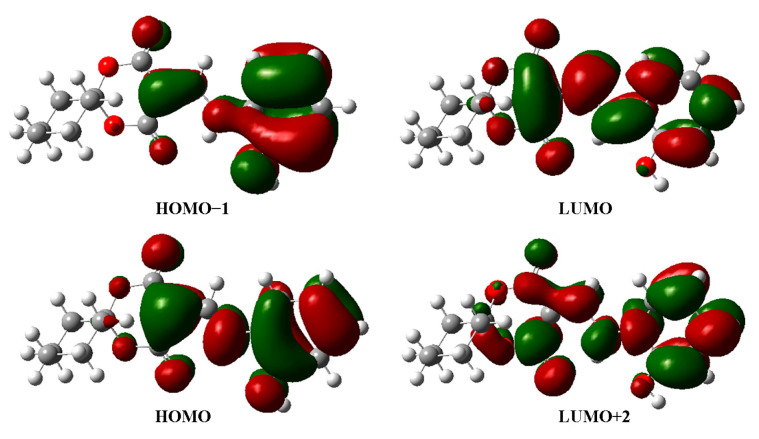
The four frontier molecular orbitals for HMD.

Furthermore, the HOMO and LUMO orbitals could be used to analyze molecular physical chemistry properties such as hardness, excitability, electron conductivity, and chemical stability (Table 5). As seen in Table 5, the calculated energies of HOMO and LUMO were −6.10 eV and −1.87 eV, respectively. A large HOMO–LUMO energy gap of 4.23 eV denoted that this molecule had weak conductivity, low reactivity, and strong stability. The HMD had a large hardness value, indicating that it was not a polarizable hard molecule. Finally, Figure 6 shows the four frontier molecular orbitals of HOMO−1, HOMO, LUMO, and LUMO+2.

### 2.4. Mulliken Population

The Mulliken population method was used to calculate the total atomic charge distributions. Mulliken was also extremely effective at detecting nucleophilic or electrophilic attacks, as well as regions sensitive to other molecular interactions. The computed charges are listed in Table 6. All hydrogen atoms had positive charges. The atom H1A coupled to atom O1 had the largest positive charge, with 0.25408. The H1 atom coupled to N1 had a charge of 0.249818, which was the second positive charge. Five atomic charge values of O1 (−0.352739), O2 (−0.348109), O3 (−0.364607), O4 (−0.316627), and O5 (−0.313277) showed high negativity, which was due to the largely electronegative state of O atoms. It was beneficial to form the N(1)–H(1)···O(3) and O(1)–H(1A)···O(2) molecular interactions.

### 2.5. Molecular Electrostatic Potential (MEP)

**Figure 7:**
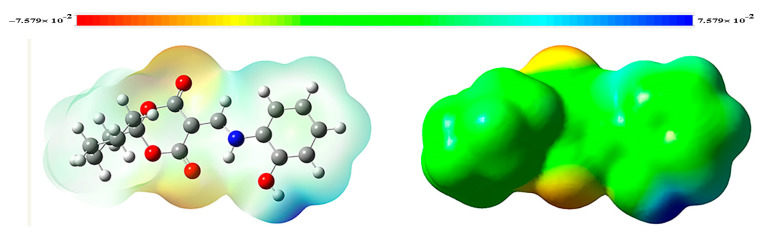
The molecular electrostatic potential for HMD.

The MEP is related to electron density and is helpful to understand the electrophilic and nucleophilic attacks as well as the hydrogen bonding interactions. The red color represents the electron-rich zones, which were suitable for the electrophilic reactions. The blue color implies the electron-poor zones, which were related to the nucleophilic reactions [22,23]. Additionally, the electrophilic and nucleophilic regions were connected by hydrogen bonds. As shown in Figure 7, the two red regions were O2 and O3 atoms bound to the carbonyl group’s C atom, which indicated that the O atom would act as an acceptor in hydrogen bonding. The blue region was located on the hydroxyl group’s H1 atom. It was demonstrated that the O(1)–H(1)···O(2) intermolecular hydrogen bonds existed in the HMD molecule (Table 3). 

### 2.6. Hirshfeld Surface Analysis

**Figure 8:**
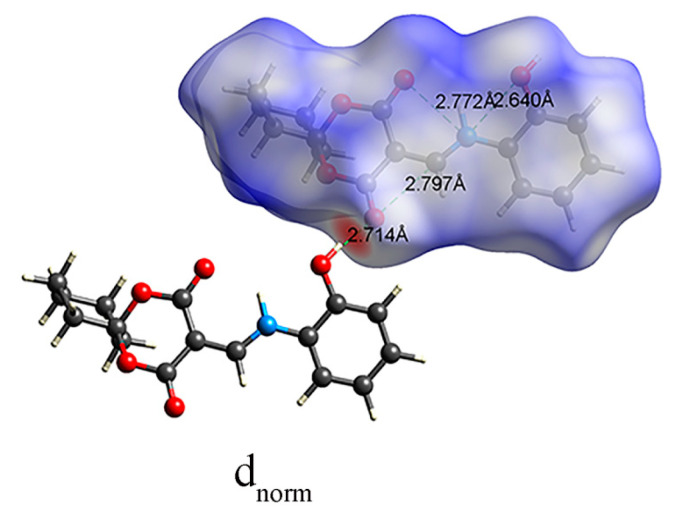
The d_norm_ Hirshfeld surface for HMD.

The Hirshfeld surface (HS) mapped with d_norm_ is depicted in Figure 8. The d_norm_-mapped HS of HMD was generated to ascertain different interactions with red, white, and blue colors. An apparent red spot in the d_norm_ surface suggested strong and short contacts, while the blue region suggested that there existed farther and weaker contacts [24,25,26]. The large red spot was detected over the O(1)–H(1A)···O(2) hydrogen bond in the molecule. The bond length (O(1)···O(2)) was 2.714 Å, and the results matched the values in the crystal.

**Figure 9:**
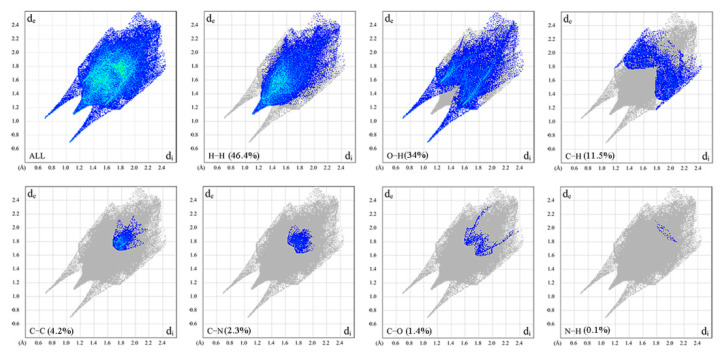
The 2D fingerprint maps for HMD.

As seen in Figure 9, the H···H interactions with a single peak composed 46.4% of the HS, which denoted the largest contribution of the total HS. The O···H/H···O interactions observed as a wing covered 34% of the total HS, whereas the C···H/H···C interactions accounted for 11.5%. Other intercontacts, including C···C, H···N/N···C, C···O/O···C, and N···H/H···N, accounted for 4.2%, 2.3%, 1.4%, and 0.1%, respectively.

### 2.7. Natural Bond Analysis (NBO)

The properties of bond orbitals and their occupation, as well as the charge transfer from the donor to the acceptor, can be analyzed by the NBO method. Delocalizing interactions between occupied and empty orbitals were decided by the stabilization energy E^(2)^. The value of E(2) was greater, indicating a more intense interaction between the donor and acceptor (Table 7).

As seen in Table 7, the intramolecular charge transfer (ICT) emerged between the π orbital and the π* orbital of the 2-hydroxyphenyl ring, which was helpful in stabilizing the molecule. For example, the stabilization energies of π (C10–C8) with π* (O3–C7) and π* (O2–C9) were 28.30, and 25.49 kcal/mol, respectively. Similarly, π (C11–C12) also showed a noticeable intramolecular interaction with π* (C13–C14) and π*(C15–C16), and their energies were 18.64 and 20.62 kcal/mol, respectively. Further, the transition π (C13–C14)→π* (C15–C16) resulted in a strong interaction energy of about 19.26 kcal/mol.

The ICT of (n→σ*/π*) could be found in N and O atoms. The interaction (n→π*) owing to the nitrogen lone pair electron donation from n (2) N1 to π* (C10–C8) emerged with the strongest energy of 62.54 kcal/mol, which was beneficial for forming the double bond. The second highest energy between n (2) N1 and π* (C11–C12) was 33.92 kcal/mol. The stabilization energies E^(2)^ of n (1) O3→σ* (N1–H1) and n (2) O3→σ* (N1–H1) were 1.98 and 6.10 kcal/mol. However, the stabilization energy between n (1) O1 and σ* (N1–H1) was 0.88 kcal/mol. This was consistent with the N–H···O intramolecular interactions of HMD.

### 2.8. Non-Linear Properties (NLO)

The mean polarizability (α), the anisotropy of polarizability (Δα), and the first hyperpolarizability parameters (β) are the basic parameters of NLO material. These values of HMD calculated using the same basis set are helpful to understand the relationships between the structure and properties.

Their equations are as follows:

$$\alpha =\frac{1}{3}\left(\alpha_{\mathrm{xx}}+\alpha_{\mathrm{yy}}+\alpha_{\mathrm{zz}}\right)$$

Δα=12[(αxx−αyy)2+(αyy−αzz)2+(αzz−αxx)2+6αxy2+6αxz2+6αzy2]12

β=[(βx2+βy2+βz2)]12

As a critical NLO material, the above three parameters of the Urea molecule had been reported as 5.07643717 × 10^−24^, 2.13568262 × 10^−24^, and 7.2228469891 × 10^−31^ esu [27]. However, the NLO parameters of HMD were computed as 320.397 × 10^−24^ esu, 276.361 × 10^−24^, and 1.94064 × 10^−30^, which were 63.11, 129.40, and 2.69 times than that of urea, respectively, as illustrated in Table 8. The high Δα and β values of HMD implied that the molecule could be used as an organic NLO material.

### 2.9. TG and DSC Analysis

**Figure 10:**
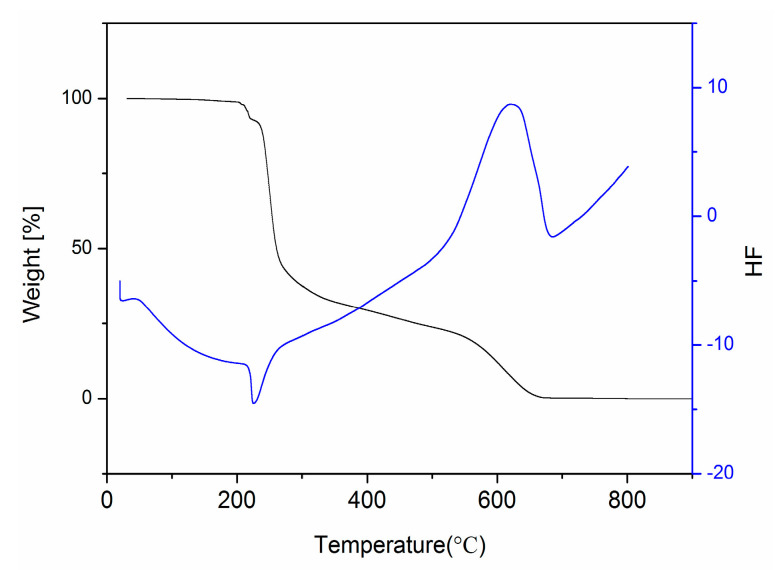
TG and DSC curves of HMD.

The thermal behavior of HMD was determined by the thermal gravimetric (TG) and differential scanning calorimeter (DSC) (Figure 10). A three-step decomposition occurred between 152 °C and 678 °C. The first weight loss of 7.2% was observed from 152 °C to 219 °C. The second weight loss of 68.9% occurred in the range of 239–262 °C. The third weight loss of 23.9% was from 262 °C to 678 °C.

One endothermic and one exothermic peak could be seen on the DSC curve. The TG curve showed no weight loss between 219 °C and 239 °C, whereas the DSC curve showed one endothermic peak at 226 °C with a starting temperature of 213 °C corresponding to its melting point. The weight (68.9%) gradually decreased after 226 °C, implying that the major groups, such as the 2-hydroxyphenylamino ring and the 1,3-dioxane ring, had decomposed. The combustion and oxidation of HMD resulted in one exothermic peak at 615 °C.

## 3. Experimental Procedure

### 3.1. General Method

The IR spectrum of HMD using KBr pellet (400–4000 cm^−1^) was measured on a Thermo Nicolet iS5 spectrophotometer. (^1^H and ^13^C) NMR was acquired using Bruker Avance III–600 in the DMSO-d_6_ solution. The TG–DSC curves were recorded using the METTLER TOLEDC tgdsc 3 in air, which heated up at a rate of 10 °C/min between 25 and 800 °C. The UV–Vis spectra were determined using a TU–1901 spectrophotometer.

### 3.2. Synthesis

The ethanol solution of 1,5-dioxaspiro[5.5]undecane-2,4-dione (0.02 mol, 3.68 g) was magnetically stirred for 3 h at 60 °C with 1.2 equivalent amount of trimethyl orthoformate (0.024 mol, 2.544 g). After that, an equal amount of o-aminophenol (0.02 mol, 2.18 g) was added, and the solution was allowed to react for another 3 h at the same temperature. The mixture was then cooled and washed with distilled water two to three times before being recrystallized with ethanol, filtered, and dried to yield the yellow powder. The yield was 21.45%. The m.p. was 213.1–213.7 °C. The ^1^H NMR (600 MHz, DMSO-d_6_) δ values (ppm) were 11.46 (s, 1H, –NH), 10.61 (s, 1H, –OH), 8.72 (s, 1H, –CH–), 6.88–7.64 (s, 4H, phenyl–H), 1.92–1.94 (m, 4H, hexamethylene–H), and 1.45–1.59 (m, 6H, hexamethylene–H). The ^13^C NMR (600 MHz, DMSO-d_6_) δ values (ppm) were 21.94, 23.69, 35.02, 86.67, 104.52, 115.77, 116.29, 119.93, 125.75, 126.66, 147.05, 151.18, 162.34, and 164.45.

### 3.3. Single Crystal Studies

A transparent yellow block crystal was measured on an Xcalibur Eos Gemini diffractometer with the Mo-Kα radiation (0.71073 Å). Its structure was achieved using SHELXT-2016 [28,29]. The H atoms of HMD were positioned at suitable positions and adjusted using a riding mode. The non-hydrogen atoms were accomplished by anisotropic refinement.

### 3.4. Theoretical Details

The optimized structure and vibrational analyses were performed with the DFT method using B3LYP/6−311G(d,p) in the Gaussian 09 package [30,31,32,33]. The electronic transition of HDM was carried out using the TD-DFT approach, which was based on the B3LYP/6−311G(d, p). The Hirshfeld surfaces and two-dimensional fingerprints were analyzed by the CrystalExplorer 17.5 [34] (Supplementary Materials).

## 4. Conclusions

Some chemical and photophysical properties of the new hydroxyphenylamin Meldrum’s acid derivative (HMD) can be understood by combining the experimental and theoretical techniques. The structural insights and vibrational spectra of the title molecule were explored using DFT calculations. The UV–Vis spectrum of HMD was compared with the experimental results. They corresponded to the π→π* and n→π* transitions of the molecule. The strong intramolecular charge transfers of π→π* and n→σ*/π* were discussed using the NBO analysis. The O(1)–H(1A)···O(2) intermolecular hydrogen bonds of the molecule could be verified by the MEP and Hirshfeld surface analysis. The large HOMO–LUMO energy gap explained the strength of the stabilization of the molecule. Finally, the NLO properties of HMD were calculated. The results showed that the molecule had a strong NLO response and might be used to design new organic NLO materials.

## Figures and Tables

**Table 1:** Experimental details of **HMD**.

Formula	C_16_H_17_NO_5_
CCDC	2233681
*Mr*	303.30
Color/shape	Yellow/block
Temperature	293(2) K
Crystal system, space group	Triclinic, *P-1*
*a*	6.9815(14) Å
*b*	8.8545(18) Å
*c*	12.707(3) Å
*α*	81.96(3)°
*β*	85.49(3)°
*γ*	70.39(3)°
*V*	732.2(3) Å^3^
*Z*	2
*D_calc_*	1.376 Mg·m^−3^
*μ*	0.103 mm^−1^
*F(000)*	320
*θ*	3.448° to 25.00°
Ranges/indices (*h, k, l*)	−8 ≤ h ≤ 8, −9≤ k ≤ 10, −13 ≤ l ≤ 15
No. of reflections collected/unique	4931/2547 [*R*_int_ = 0.0359]
No. of parameters	199
GOF	1.031
*R*_1_ [*I* > 2*σ*(*I*)]	0.0571
*wR*_2_ [*I* > 2*σ*(*I*)]	0.1345
*R*_1_ [all data]	0.0896
*wR*_2_ (all data)	0.1628
Largest diff. peak and hole	0.217 e. Å^−3^and −0.245 e. Å^−3^

**Table 2:** The selected experimental and B3LYP parameters of HMD.

Geometrical Parameters	Exp	B3LYP
Bond length (Å)		
N(1)–C(10)	1.321(3)	1.333
N(1)–C(11)	1.413(3)	1.408
C(8)–C(10)	1.385(3)	1.380
C(16)–O(1)	1.354(3)	1.365
O(3)–C(7)	1.220(3)	1.217
O(2)–C(9)	1.221(3)	1.206
Bond angle (°)		
C(10)–N(1)–C(11)	125.90(2)	126.85
N(1)–C(10)–C(8)	125.70(2)	125.38
C(10)–C(8)–C(7)	122.40(2)	121.22
C(10)–C(8)–C(9)	117.00(2)	117.39
C(12)–C(11)–N(1)	124.00(2)	124.12
C(16)–C(11)–N(1)	116.50(2)	116.87
Torsion angle (°)		
C(11)–N(1)–C(10)–C(8)	−175.50(2)	−178.03
C(7)–C(8)–C(10)–N(1)	−2.30(3)	−0.88
C(9)–C(8)–C(10)–N(1)	−175.60(2)	−175.85

**Table 3:** The hydrogen bonds for HMD.

D–H···A	Symmetry	D–H (Å)	H…A(Å)	D…A(Å)	∠D–H···A(°)
N(1)–H(1)···O(3)	Intra	0.86	2.1333	2.772(3)	130.68
O(1)–H(1A)···O(2)	−1 + *x*, 1 + *y*, *z*	0.82	1.8974	2.714(2)	173.65

**Table 4:** Experimental and calculated electronic transition parameters for HMD.

Exp.	Calc. (TD-DFT)		
Wavelength (nm)	Wavelength (nm)	Oscillator Strength (f)	Electronic Transition Orbits
206	191	0.1113	79HOMO−1→83LUMO+2 (40.64%)
234	226	0.1123	80HOMO→83LUMO+2 (48.30%)
346	321	0.6683	80HOMO→81LUMO (97.0%)

**Table 5:** Global descriptor values for HMD.

Parameters (eV)	HMD
E_HOMO_	−6.10
E_LUMO_	−1.87
Energy gap Δ*E*	4.23
Ionization potential (*I* = −E_HOMO_)	6.10
Electron affinity (*A* = −E_LUMO_)	1.87
Global hardness (*η* = (*I* − *A*)/2)	2.11
Global softness (*ζ* = 1/2*η*)	0.236
Chemical potential (*μ* = − (*I* + *A*)/2)	−4.285
Global electrophilicity (*ω* = *μ*^2^/2*η*)	4.645
Electron negativity (*χ* = (*I* + *A*)/2)	4.285
Maximum charge transfer index (Δ*N_max_.* = −*μ*/*η*)	2.031

**Table 6:** The B3LYP-computed Mulliken atomic charges (in a.u.) for HMD.

Atom	Charge	Atom	Charge	Atom	Charge	Atom	Charge
O5	−0.313277	O1	−0.352739	C12	−0.057529	C2	−0.207575
O4	−0.316627	H1A	0.254086	H12	0.100675	H2A	0.110945
N1	−0.385915	C11	0.160787	C14	−0.066608	H2B	0.121645
H1	0.249818	C9	0.441547	H13	0.087573	C6	−0.188236
C8	−0.485979	C15	−0.062515	C4	0.097509	H6A	0.117361
O3	−0.364607	H15	0.085894	C14	−0.085738	H6B	0.111042
O2	−0.348109	C3	−0.097413	H13	0.087020	C1	−0.216698
C16	0.090027	H3A	0.121878	C5	−0.115944	H1A	0.110428
C10	0.269087	H38	0.117636	H5A	0.126228	H1C	0.102233
H10	0.104773	C7	0.484310	H5B	0.113007		

**Table 7:** The stabilization energy E^(2)^ for HMD.

Donor NBO (i)→Acceptor NBO (j)	E^(2)^ kcal/mol	Donor NBO (i)→Acceptor NBO (j)	E^(2)^ kcal/mol
π(C10–C8)→π* (O3–C7)	28.30	n (2) O1→π* (C15–C16)	27.74
π(C10–C8)→π* (O2–C9)	25.49	n (2) O3→σ*(O4–C5)	31.10
π(C11–C12)→π* (C15–C16)	20.62	n (2) O2→σ* (O5–C9)	33.73
π (C11–C12)→π* (C13–C14)	18.64	n (2) N1→π* (C10–C8)	62.54
π (C13–C14)→π* (C15–C16)	19.26	n (2) N1→π* (C11–C12)	33.92
n (1) O3→σ* (N1–H1)	1.98	n (2) O5→π* (O2–C9)	29.40
n (2) O3→σ* (N1–H1)	6.10	n (2) O4→π* (O3–C7)	33.74
n (1) O1→σ* (N1–H1)	0.88		

**Table 8:** The NLO parameters of HMD.

Parameters	Value (esu)
μ_x_	3.3257
μ_y_	−2.4746
μ_z_	1.7942
μ	4.5170
α_xx_	501.183 × 10^−24^
α_yy_	278.439 × 10^−24^
α_zz_	181.569 × 10^−24^
α_xy_	−0.42461 × 10^−24^
α_xz_	2.12344 × 10^−24^
α_yz_	1.70112 × 10^−24^
α	320.397 × 10^−24^
Δα	276.361 × 10^−24^
β_x_	1.297049 × 10^−30^
β_y_	0.903273 × 10^−30^
β_z_	1.125 × 10^−30^
β	1.94064 × 10^−30^

## Data Availability

Not applicable.

## Supplementary Materials

The following supporting information can be downloaded at: https://www.mdpi.com/article/10.3390/molecules28052181/s1, file S1: checkCIF/PLATON report.

## Sample Availability

Samples of the compounds are not available from the authors.
